# Preclinical Evaluation of BMP-9-Treated Human Bone-like Substitutes for Alveolar Ridge Preservation following Tooth Extraction

**DOI:** 10.3390/ijms23063302

**Published:** 2022-03-18

**Authors:** Fabien Kawecki, Jessica Jann, Michel Fortin, François A. Auger, Nathalie Faucheux, Julie Fradette

**Affiliations:** 1 Centre de Recherche en Organogénèse Expérimentale de l’Université Laval, LOEX, Division of Regenerative Medicine, CHU de Québec Research Center-Université Laval, Quebec City, QC G1V 0A6, Canada; fabien.kawecki.1@ulaval.ca (F.K.); michel.fortin@fmd.ulaval.ca (M.F.); francois.auger@fmed.ulaval.ca (F.A.A.); 2 Department of Surgery, Faculty of Medicine, Université Laval, Quebec City, QC G1V 0A6, Canada; 3 Clinical Research Center of CHU de Sherbrooke, Department of Chemical and Biotechnological Engineering, Pharmacology Institute of Sherbrooke, Université de Sherbrooke, Sherbrooke, QC J1H 5N4, Canada; jessica.jann@usherbrooke.ca (J.J.); nathalie.faucheux@usherbrooke.ca (N.F.); 4 Faculty of Dentistry, Université Laval, Quebec City, QC G1V 0A6, Canada; 5 Service of Oral and Maxillofacial Surgery, CHU de Québec-Université Laval, Quebec City, QC G1V 0A6, Canada

**Keywords:** bone, mesenchymal stem cells, bone morphogenetic protein-9, tissue engineering, dental biomaterial

## Abstract

The success of dental implant treatment after tooth extraction is generally maximized by preserving the alveolar ridge using cell-free biomaterials. However, these treatments can be associated with inflammatory reactions, leading to additional bone volume loss hampering dental implant positioning. Our group developed a self-assembled bone-like substitute constituted of osteogenically induced human adipose-derived stromal/stem cells (hASCs). We hypothesized that a bone morphogenetic protein (BMP) supplementation could improve the in vitro osteogenic potential of the bone-like substitute, which would subsequently translate into enhanced alveolar bone healing after tooth extraction. ASCs displayed a better osteogenic response to BMP-9 than to BMP-2 in monolayer cell culture, as shown by higher transcript levels of the osteogenic markers *RUNX2*, *osterix* (*OSX*/*SP7*), and *alkaline phosphatase* after three and six days of treatment. Interestingly, BMP-9 treatment significantly increased *OSX* transcripts and alkaline phosphatase activity, as well as pro-angiogenic *angiopoietin-1* gene expression, in engineered bone-like substitutes after 21 days of culture. Alveolar bone healing was investigated after molar extraction in nude rats. Microcomputed tomography and histological evaluations revealed similar, or even superior, global alveolar bone preservation when defects were filled with BMP-9-treated bone-like substitutes for ten weeks compared to a clinical-grade biomaterial, with adequate gingival re-epithelialization in the absence of resorption.

## 1. Introduction

Tooth extraction may be necessary for several reasons such as periodontal diseases, tooth decay, and dento-alveolar trauma, among others [1]. Clinical studies have demonstrated that alveolar ridge bone resorption is initiated immediately after tooth removal in the vertical and horizontal planes [2,3,4]. This process leads to an average of 40 to 60% bone loss during the first two years after tooth extraction [5]. Furthermore, the resorption rate is significantly elevated in the maxillary bone during the first six months post-extraction period [6]. Since the alveolar ridge is damaged after a dental extraction, bone tissue preservation is required to optimize dental implant positioning. Various materials are used in dentistry for alveolar ridge preservation, including autografts, demineralized freeze-dried human (allografts) or bovine (xenografts) bone (DFDBA), mineralized freeze-dried bone allografts, inorganic synthetic or bovine hydroxyapatite (HA), or synthetic biomaterials [4,7,8,9,10]. Even if these graft materials can preserve alveolar ridge dimensions to some extent, the quality and the quantity of new bone tissue that may form in the alveolar socket vary between patients [11,12]. In addition, these biomaterials may cause inflammatory reactions, foreign body responses, and/or fibrous encapsulation once grafted [13].

Promising options involving bone tissue engineering have been recently developed in the field of dental and oral reconstruction [14]. In general, tissue-engineered substitutes combine human cells with biomaterial scaffolds and growth factors to produce native-like substitutes [15,16]. Growth factors play an important role in the maturation and differentiation processes of the cells composing the bone substitutes. In particular, the use of several bone morphogenetic proteins (BMPs) from the transforming growth factor-ß (TGF-ß) superfamily was granted permission for the treatment of spinal fusion, long-bone fractures, long-bone non-unions, and dental implant placement in humans [17,18,19]. In the context of oral surgery, recombinant human BMP-2 (rhBMP-2) loaded with either deproteinized bovine bone mineral blocks (Geistlich Bio-Oss^®^) or autogenous bone blocks has been shown to favor primary ridge augmentation in patients four months after implantation [20]. In addition, it was revealed that BMP-9 is widely expressed in odontoblasts, ameloblasts, dental pulp cells, and osteoblasts in alveolar bones [21]. It has been shown that rhBMP-9 combined with biomaterials, such as Geistlich Bio-Oss^®^ blocks or Geistlich Bio-Gide^®^ collagen barrier membranes, positively induced bone formation in a guided bone regeneration rabbit model eight weeks after implantation [22]. Therefore, BMPs represent an interesting option for ridge augmentation.

Over the years, our research team has developed all-natural bone-like substitutes based on cell-assembled extracellular matrix components surrounding live osteogenically induced human adipose-derived stromal/stem cells (hASCs) [23,24,25]. The capacity to differentiate towards the osteogenic lineage had already been established for this mesenchymal cell population [26]. We hypothesized that rhBMP-2 or rhBMP-9 treatments during the bioproduction could improve in vitro osteogenesis of hASCs within the engineered substitutes and ultimately favor in vivo alveolar bone preservation after tooth extraction in a nude rat model.

## 2. Results

### 2.1. Better Osteogenic Differentiation Is Achieved When Monolayer Cultures of Osteogenically Induced hASCs Are Treated with rhBMP-9 Compared with rhBMP-2

**Figure 1:**
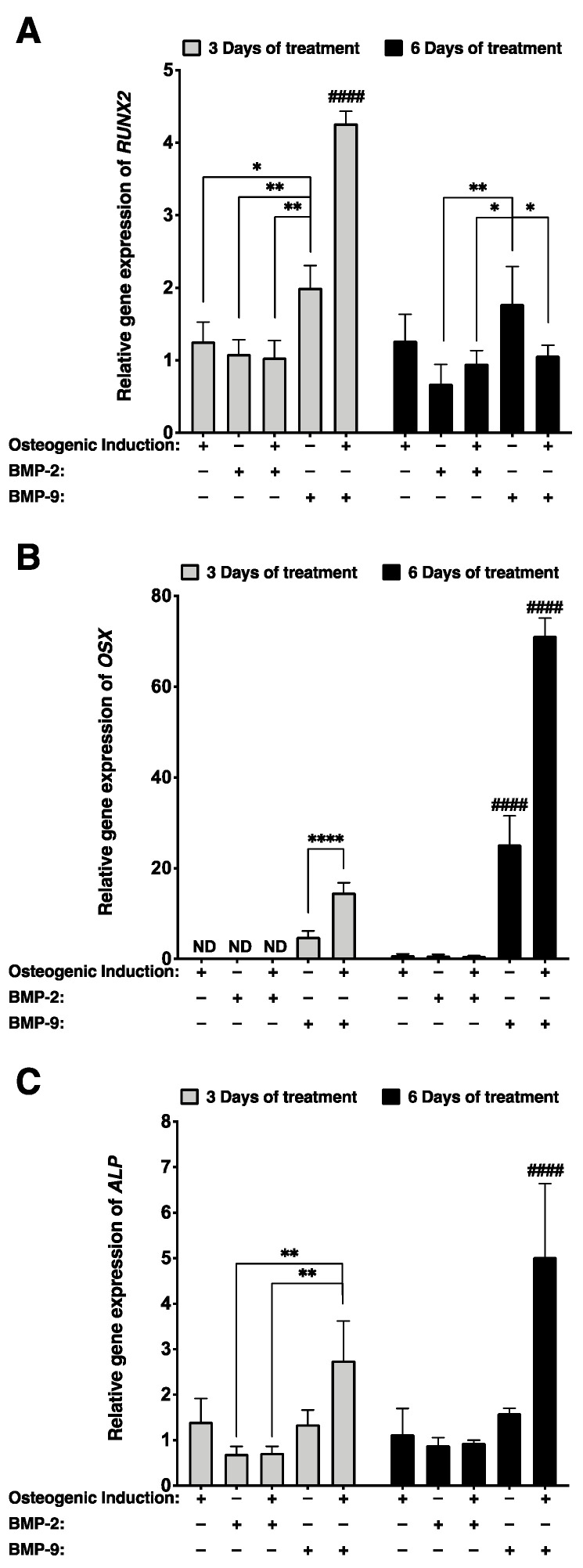
Robust osteogenic gene expression profiles from osteogenically induced hASCs when treated with rhBMP-9 in 2D cell culture. (**A**–**C**) Relative gene expression of (**A**) *runt-related transcription factor 2* (*RUNX2*), (**B**) *osterix* (*OSX*), and (**C**) *alkaline phosphatase* (*ALP*) measured by RT-qPCR on osteogenically induced and non-induced hASCs treated with 1 nM of rhBMP-2 or rhBMP-9 for three or six days. (Two-way ANOVA with a Bonferroni’s multiple comparison post-hoc test; significant differences between two conditions per time point: * *p* < 0.05, ** *p* < 0.01, **** *p* < 0.0001 and significant difference between one condition and all other conditions: ^####^ *p* < 0.0001).

Osteogenic differentiation of hASCs treated for three and six days with 1 nM rhBMP-2 or 1 nM rhBMP-9 was assessed by determining *runt-related transcription factor 2* (*RUNX2*), *osterix* (*OSX*), and *alkaline phosphatase* (*ALP*) gene expression in 2D cell cultures using RT-qPCR. Gene expressions for each group were referred to the untreated non-induced stromal group (Figure 1). Results showed that in 2D cell culture, the *RUNX2* transcript of hASCs remained unchanged despite osteogenic induction (gene expressions relative to non-induced hASCs approximate the value of one; Figure 1A). However, hASCs treated for three days with rhBMP-9 in the absence of osteogenic medium displayed more elevated *RUNX2* gene expression compared with either an osteogenic induction alone (1.6-fold; * *p* < 0.05) or a rhBMP-2 treatment (1.9-fold; ** *p* < 0.01) (Figure 1A). In addition, significantly higher *RUNX2* gene expression was measured at the early stage of culture (after three days of treatment) when osteogenically induced hASCs were treated with rhBMP-9 compared with rhBMP-2 (4.1-fold; ^####^ *p* < 0.0001) or compared with cells induced with osteogenic medium alone (3.4-fold; ^####^ *p* < 0.0001) (Figure 1A). *RUNX2* transcripts of osteogenically induced hASCs treated for six days with rhBMP-9 drastically decreased, suggesting that the cells are at a later stage of osteogenesis.

At the early stage of culture, *OSX* gene expression was only detectable when hASCs were treated with rhBMP-9 for three days, and the association of this treatment with osteogenic induction significantly increased transcript levels (3-fold; **** *p* < 0.0001) (Figure 1B). A similar trend was observed after six days of treatment since low levels of *OSX* gene expression were detected in the absence of rhBMP-9 treatment and osteogenic induction associated with rhBMP-9 significantly upregulated gene expression (2.8-fold; ^####^ *p* < 0.0001) (Figure 1B).

After three days of treatment, *ALP* gene expression was significantly more elevated in osteogenically induced hASCs treated with rhBMP-9 than with rhBMP-2 (3.8-fold; ** *p* < 0.01) (Figure 1C). In addition, *ALP* gene expression was considerably increased after six days of culture and became significantly higher than for other conditions (^####^ *p* < 0.0001) (Figure 1C). These results showed that an osteogenic induction protocol including a 1 nM rhBMP-9 supplementation improves the osteogenesis of hASCs in 2D cell culture. In addition, these results suggest that rhBMP-9 is a better candidate than rhBMP-2 for the production of bone-like substitutes composed of osteogenically induced hASCs.

### 2.2. Increased Osteogenic Differentiation When Bone-like Substitutes Are Produced with Concomitant rhBMP-9 Supplementation

**Figure 2:**
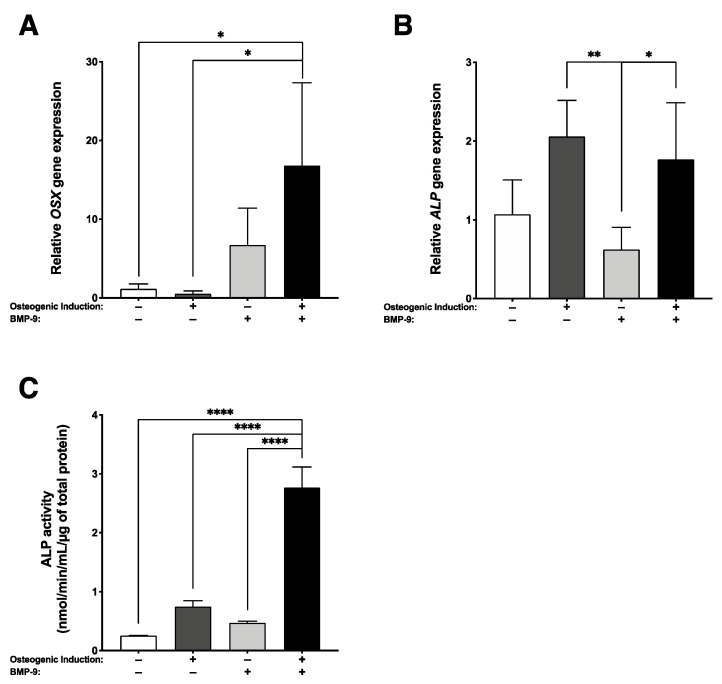
Superior osteogenic profile of the BMP-9 treated bone-like substitutes. Gene expression of (**A**) *osterix* (*OSX*) and (**B**) *alkaline phosphatase* (*ALP*) were measured by RT-qPCR on substitutes cultured for 21 days. (**C**) For osteogenically induced substitutes treated with 1 nM of rhBMP-9, *ALP* activity was higher compared to other conditions. (One-way ANOVA with a Bonferroni’s multiple comparison post-hoc test, * *p* < 0.05, ** *p* < 0.01, **** *p* < 0.0001).

The pro-osteogenic gene expression profiles of 3D substitutes cultured for 21 days were also assessed using RT-qPCR. A sustained rhBMP-9 treatment associated with osteogenic induction during the engineering of self-assembled bone-like substitutes significantly increased *OSX* gene transcript (33.6-fold; * *p* < 0.05) (Figure 2A). While bone-like substitutes supplemented or not with rhBMP-9 showed similar *ALP* gene expression, expression levels were significantly more elevated than for stromal substitutes treated with rhBMP-9 (3.3-fold; ** *p* < 0.01 and 2.8-fold; * *p* < 0.05, respectively) (Figure 2B). Herein, *ALP* activity was measured in the media conditioned for 48 h by engineered substitutes, prior to in vivo implantation (21 days of culture). Results show that an rhBMP-9 treatment associated with an osteogenic induction for the engineering of bone-like substitutes significantly enhanced *ALP* activity compared to other culture conditions (**** *p* < 0.0001) (Figure 2C). These results support that an osteogenic induction protocol including supplementation with 1 nM BMP-9 favors the osteogenesis of the hASC-derived substitutes during engineering.

### 2.3. Increase in Angiopoietin-1 Gene Expression When Bone-like Substitutes Are Treated with rhBMP-9

**Figure 3:**
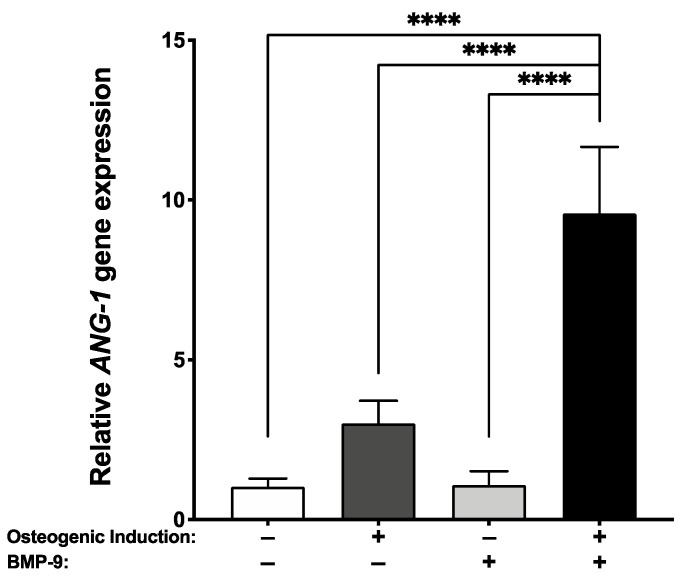
Superior expression of *angiopoietin-1* transcript for the BMP-9 treated bone-like substitutes. Gene expression of *angiopoietin-1* (*ANG-1*) was measured by RT-qPCR after 21 days of tissue culture. (One-way ANOVA with a Bonferroni’s multiple comparison post-hoc test, **** *p* < 0.0001).

After 21 days of tissue culture, the pro-angiogenic *ANG-1* gene transcript levels were evaluated by RT-qPCR. Results show that an rhBMP-9 treatment associated with an osteogenic induction for the engineering of bone-like substitutes significantly increases *ANG-1* gene expression compared to other culture conditions (**** *p* < 0.0001) (Figure 3). These data suggest that exposure to an osteogenic induction media supplemented with 1 nM rhBMP-9 changed every two/three days for 18 days (21 days of culture) could favor a pro-angiogenic profile of the hASC-derived substitutes.

### 2.4. Microcomputed Tomography Imaging and Analysis of the Alveolar Bone Tissue Preservation

**Figure 4:**
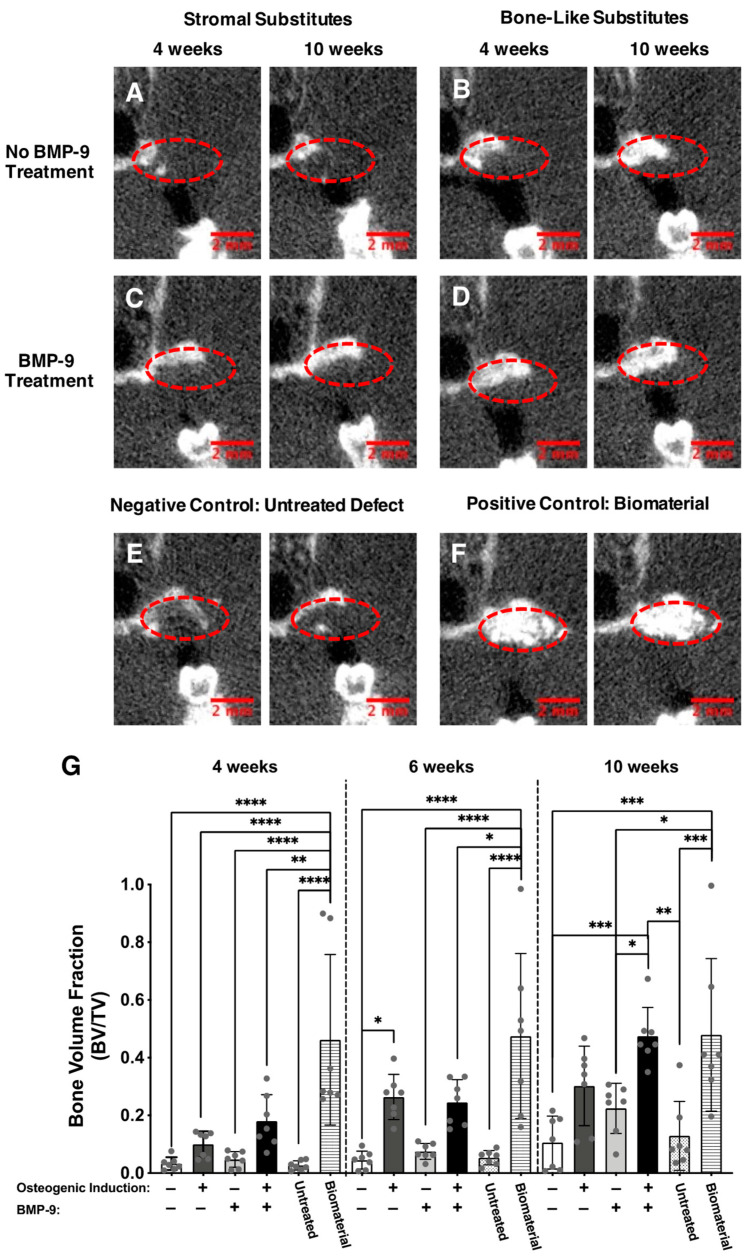
Microcomputed tomography imaging and analysis of the implantation sites. (**A**–**F**) Representative coronal plane images of the implantation sites (red dotted circles) showing alveolar bone preservation (white) following tooth extraction either four and ten weeks after grafting. Scale bars: 2 mm. (**G**) The ratio of bone volume (BV) reported on the tissue volume (TV), also named bone volume fraction (BVF), was calculated at the implantation site four, six, and ten weeks after implantation. Each gray dot represents an implantation site. (Two-way ANOVA with a Bonferroni’s multiple comparison post-hoc test, * *p* < 0.05, ** *p* < 0.01, *** *p* < 0.001, **** *p* < 0.0001).

To evaluate the alveolar bone healing capacity of the various engineered substitutes, microcomputed tomography scans were performed on the same anesthetized animals at four, six, and ten weeks after implantation. Coronal plane images of the animal’s heads (Figure 4A–F) showed elevated new bone formation (within dotted areas) when alveolar bone defects were filled for ten weeks with either rhBMP-9-treated bone-like substitutes (Figure 4D) or a clinical-grade biomaterial (Figure 4F). These observations were confirmed with bone volume fraction (BVF) measurements of the areas where the implantation of the substitutes was performed (Figure 4G). Globally, all engineered substitutes and untreated defects showed BVF augmentation over time at the extraction sites. This was clearly observed for the BMP-9-treated bone-like substitute group, which exhibited a linear BVF increase over time (Figure S1A; linear regression; R^2^ = 0.84) and reached the mean level of the group grafted with the clinical-grade biomaterial after ten weeks. Indeed, relatively similar levels were observed between the BMP-9-treated bone-like substitute (BVF = 0.4743) and the clinical-grade biomaterial (BVF = 0.4789) after ten weeks (Figure 4G and Figure S1B). After ten weeks of alveolar bone healing, BMP-9-treated bone-like substitutes showed more elevated BVT compared to untreated (4.5-fold; *** *p* < 0.001) or BMP-9-treated (2.1-fold; * *p* < 0.05) stromal substitutes, and defects left empty (3.7-fold; ** *p* < 0.01) (Figure 4G). BVF results were similar for untreated and BMP-9-treated bone-like substitutes using two-way ANOVA with a Bonferroni’s multiple comparison post-hoc test on the complete set of data including all groups. However, statistical analysis focusing on the untreated and BMP-9-treated bone-like substitute groups’ data sets revealed a significant increase when the animals were grafted for ten weeks with substitutes that were osteogenically induced and treated with rhBMP-9 (1.6-fold; ** *p* < 0.01) (Figure S1A). The biomaterial group displayed important variability in terms of bone healing, and this was demonstrated by elevated coefficients of variation at Week 4 (63.9%), Week 6 (60.3%), and Week 10 (55.2%). Furthermore, several animals grafted with this biomaterial showed resorption of the graft over time (Figure S2). These results suggest that BMP-9-treated bone-like substitutes represent a promising bone filler for alveolar ridge preservation following tooth extraction.

### 2.5. Gingival Healing

Qualitative assessment of gingival healing was performed ten weeks after tooth extraction before proceeding with the terminal tissue analyses. While no systematic macroscopic imaging follow-ups were realized over time (four, six, and ten weeks), no severe inflammatory and adverse reactions were observed post-operatively on the anesthetized animals during the microcomputed tomography imaging. After ten weeks of implantation, untreated defects, as well as BMP-9-treated stromal substitutes, showed a low rate of re-epithelialization (42.9% and 28.6%, respectively) with the presence of dehiscence or graft exposure (Figure S3A). Elevated re-epithelialization rates were observed when the implantation sites were grafted with bone-like substitutes treated or not with BMP-9 (both 85.7%) (Table 1 and Figure S3B). Gingival healing was considered sufficient when the percentage of re-epithelialization was superior to 85%. These results indicate that, independently of BMP-9 treatment, the bone-like substitutes are interesting candidates to favor gingival healing after tooth extraction.

### 2.6. Improved Alveolar Bone Healing Observed following Histological Analyses

**Figure 5:**
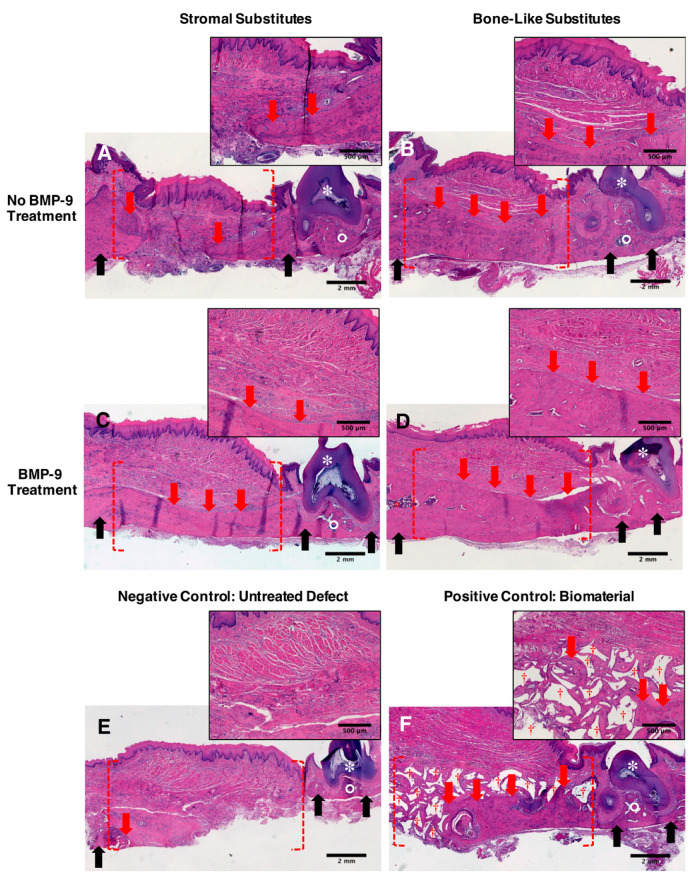
Representative histological observations of the grafting sites. (**A**–**F**) Maxillaries were harvested, fixed, demineralized, paraffin-embedded, and stained using hematoxylin and eosin dyes ten weeks after grafting of the substitutes. Implantation sites (red dashed parentheses) can be identified next to the third superior molar (white asterisks; color: dark purple). The area containing the remaining tooth is delimited by native bone (black arrows; color: purple) surrounding bone marrow (white circle color: dark purple). The new bone that is formed (red arrows; color: purple) is surrounded by dense irregular connective tissue (color: pink) and observed at the grafting sites. (**F**) The presence of empty spaces (red crosses) surrounded by newly formed bone corresponds to the presence of the demineralized biomaterial granules and was observed in the positive control group. Scale bars: 2 mm (large panels) and 500 µm (insets).

Alveolar bone healing was evaluated ten weeks after surgery on cross-sections on tissues from the implantation sites stained with hematoxylin and eosin dyes. Stromal substitutes without rhBMP-9 treatment (Figure 5A) and untreated groups (Figure 5E) showed low levels of new bone formation (observable by the presence of purple-colored dense and regular connective tissue) with incomplete regeneration of the bone defects after ten weeks. The BMP-9-treated stromal substitute group revealed a moderate formation of new bone (purple-colored dense and regular connective tissues in Figure 5C). An elevated alveolar bone healing was observed in both the bone-like substitutes supplemented or not with rhBMP-9 treatment (purple-colored dense and regular connective tissues in Figure 5B,D). Observations of the edges of the initial defect showed great integration of the substitutes. On the other hand, histological observations of the biomaterial group showed moderate new bone formation associated with the presence of void spaces (Figure 5F). These empty spaces in histological sections likely correspond to the disappearance of biomaterial granules dissolved during the demineralization step before embedding and staining. Altogether, these results validate the results obtained by microcomputed tomography.

## 3. Discussion

BMPs are widely known for their role in bone regeneration [27,28,29,30]. This study evaluated the osteogenic effect of a BMP supplementation during the engineering of self-assembled bone-like substitutes using osteogenically induced hASCs in vitro and the associated potential for alveolar bone healing in vivo. BMP-2 and -9 growth factors are members of the TGF-β superfamily [31,32]. Similar to other BMPs, they activate the Smad-signaling pathway after binding BMP receptors [33] and initiate osteogenic gene expression by the promotion of pro-osteogenic transcription factors such as *RUNX2*, *OSX*, or functional genes such as *ALP* in various stem cells (rabbit ASCs, C3H10T1/2 mesenchymal cells, C2C12 murine multilineage cells, or mouse embryonic fibroblasts) [34,35,36,37]. Runx2 transcription factor plays an essential role in the engagement of stem cells/osteoprogenitors towards the osteochondral differentiation pathway, whereas the osterix transcription factor mainly acts on the terminal phases of osteoblastic differentiation [38,39]. The *ALP* gene is highly expressed by osteoblast cells and is considered an early osteoblastic marker [40].

The osteogenic effect of BMP-9 has been historically established using immortalized murine ASCs [41]. In this study, we show that human postnatal ASCs have a better osteogenic response when exposed to 1 nM of rhBMP-9 than 1 nM of rhBMP-2 in 2D cell culture. This osteogenic induction was reflected by a significant expression of the pro-osteogenic genes *RUNX2*, *OSX*, and *ALP*. Our findings are consistent with a study by Rivera et al. showing that hASCs have higher *ALP* activity under BMP-9 than BMP-2 treatment [42].

Using recombinant adenoviruses expressing fourteen human BMPs, the research group of Dr. He found that BMP-9 is one of the most potent osteoinductive factors [27,43]. The fact that BMP-9 was not affected by Noggin, an extracellular inhibitor of BMPs such as BMP-2, might explain such osteoinductive potential [44]. Our previous studies also showed that both murine and human muscle resident stromal cells (CD31(−) CD34(−) CD90(−) CD73(+) CD105(+) CD140(−) or murine preosteoblasts strongly respond to rhBMP-9 compared with rhBMP-2 in the presence of fetal bovine serum (FBS) [45,46,47]. For example, based on *ALP* activity measurement, the half-maximum effective concentration (EC50) for rhBMP-9 treatment on human muscle resident mesenchymal stromal cells (hmrSCs) in FBS presence is approximately 40-fold lower compared to rhBMP-2 and 12 times more rhBMP-2 is required to reach the plateau compared with rhBMP-9 (plateau at 1 nM) [45]. Interestingly, human hematoma from fractures and serum from healthy human donors can also potentiate the hmrSC response to rhBMP-9 drastically in terms of osteogenic differentiation [45].

Based on their sequence homology, BMP-2 and BMP-9 are not classified in the same BMP subfamily, and they also do not use the same Ser/Thr kinase type I receptors [31]. BMP-9 interacts with activin receptor-like kinase (ALK) 1 (high affinity) and ALK2, while BMP-2 forms with ALK3 a thermodynamically more stable complex than BMP-9/ALK3 in aqueous solution [31,48,49]. BMP-9 and BMP-2 can induce different mitogen-activated protein kinases cascades activation. For example, the extracellular-signal-regulated kinase 1/2 pathway inhibition and increased p38 phosphorylation in MC3T3-E1 preosteoblasts were suggested to explain the effect of serum on preosteoblast response to rhBMP-9 compared with rhBMP-2 [47]. In addition, the costs being similar between rhBMP-9 and -2, combined with the better osteogenic response of hASCs to BMP-9 compared to BMP-2, suggest a high clinical potential of the treatment proposed in this study. Further studies are required to understand better how BMP-2 and BMP-9 transduce their signal in hASCs.

We thus selected rhBMP-9 to promote osteogenesis within hASC-derived 3D bone-like substitutes, with the aim to achieve more efficient in vivo alveolar bone preservation after tooth extraction. In vitro, the long-term rhBMP-9 supplementation (18 days) of hASC-based bone-like substitutes during their bioproduction (21 days) resulted in a significant increase in osteogenic markers. The cell-secreted *ALP* enzyme is a standard marker used to determine osteogenesis induction level in several mesenchymal cells since it allows the release of phosphate ions from matrix vesicles that are subsequently associated with calcium ions to form hydroxyapatite crystals [50,51]. Indeed, the combined detection of elevated *OSX* and *ALP* gene transcripts and *ALP* enzyme activity suggests that hASCs have reached an advanced stage of osteogenic differentiation in vitro.

To evaluate the alveolar bone preservation potential of the substitutes, engineered tissues were grafted into defects generated after tooth extraction. The positive control group was composed of animals grafted with the clinical-grade BoneCeramic^TM^ biomaterial. This biomaterial already showed effectiveness for calvarial bone defect reconstruction [52] and alveolar bone defect preservation during tooth movement in rats [53]. A clinical trial including thirty patients suffering from posterior edentulous maxillary defects and vertical bone reductions established that a treatment involving this biomaterial provided similar histological and radiological outcomes compared to autologous bone graft [54]. Therefore, we used this group as a reference although our experimental design did not include the use of an overlying membrane placement as usually performed in a clinical setting.

Our results support that, compared to clinical-grade biomaterial, hASC-derived bone-like substitutes treated in vitro with 1 nM of rhBMP-9 display similar, or even superior, alveolar bone preservation potential following tooth extraction with low variability of bone volume fraction, no sign of resorption, and a stable alveolar bone healing over time (Figure S1A). In addition, the rhBMP-9-treated bone-like substitute showed interesting outcomes of gingival healing, thus suggesting that these new alveolar bone fillers may be as efficient as the clinical-grade biomaterial, which was more exposed to resorption in our study (Figure S2).

Other research groups developed different BMP-9-based strategies for the treatment of bone defects. For example, Nie et al. reported the use of a scaffold composed of coralline hydroxyapatite associated with rat dental follicle stem cells (DFCs) transfected with a recombinant adenoviral vector to overexpress BMP-9 [55]. Briefly, they subcutaneously implanted the scaffolds including DFCs that were transduced with an adenovirus expressing BMP-9, or not, in the *dorsa* of immunodeficient mice. Higher bone volume fractions were measured by micro-CT when the scaffolds were cellularized with untransduced and transduced DFCs compared to the scaffold alone. Additionally, elevated ectopic bone formation was observed when scaffolds were seeded with BMP-9-transduced DFCs compared to control DFCs. More recently, Freitas et al. proposed a cell therapy using genetically edited immortalized bone-marrow MSCs to overexpress BMP-9 using CRISPR-Cas9 technology to favor in vivo bone formation in rat calvarial bone defects [56]. Their micro-CT results clearly showed that genetically modified cells injected within the defects enhanced new bone formation. However, these types of approaches are complex and require careful considerations since the transduction or gene editing processes may remain difficult to control in vivo for long-term bone formation. In this study, we propose a self-assembly approach of tissue engineering that uses a more basic construct composed of a completely biological material composed of human cell-assembled extracellular matrix components. In addition, this bioinspired material includes living human ASCs that were osteogenically induced, but not genetically modified, and treated with a precise concentration of 1 nM rhBMP-9 in vitro until implantation. While rhBMP-9 can be trapped within the biomaterial during its bioproduction, the quantity of molecules released should be low and short-term once implanted. This type of molecular action makes the biomaterial safer than one containing genetically modified cells that continuously secrete BMP-9 molecules.

We previously showed that a traditional osteogenic induction cocktail can enhance the transcription and protein secretion of *angiopoietin-1* by hASCs [24]. The current results indicated that rhBMP-9 supplementation to the same osteogenic media further increased the expression of *angiopoietin-1* gene transcript. The *angiopoietin-1* (*ANG-1*) protein promotes in vitro angiogenesis by stimulating endothelial cell migration, tubule-like structure formation, and in vivo neovascularization [57,58,59]. These pro-angiogenic properties are expected to enhance therapeutic outcomes when substitutes are implanted in vivo, in particular by promoting graft neovascularization that would favor bone healing [57,58,59]. Since we previously showed in a rodent calvarial bone defect model that prevascularized bone-like substitutes improved the graft survival after 12 weeks of implantation [25], we hypothesize that adding a rhBMP-9 treatment to our protocols would allow the production of a prevascularized bone-like substitute with superior in vivo bone healing level and graft survival.

## 4. Materials and Methods

### 4.1. Human Adipose-Derived Stromal/Stem Cell Isolation, and Expansion

Human ASCs were isolated from a donor (informed written consent was obtained from a healthy female donor, age: 35, body mass index: 21.0 (weight in kilograms divided by the square of the height in meters)) according to our previously described protocols [60], following subcutaneous adipose tissue lipoaspiration procedures. For the monolayer cultures with BMPs, as well as before the production of the tissue-engineered substitutes, cryopreserved hASCs (Passages 5 and 6, respectively) were first expanded in Dulbecco’s modified Eagle’s medium (DMEM) supplemented with 10% fetal bovine serum (FBS) (Wisent Bioproducts, St-Bruno, QC, Canada) and antibiotics (100 U/mL penicillin (Sigma-Aldrich, Oakville, ON, Canada) and 25 g/mL gentamicin (Schering-Plough Canada Inc./Merck, Scarborough, ON, Canada)) and cultured in a humidified 37 °C incubator with 8% CO_2_.

### 4.2. Monolayer Cell Culture and BMP Response Assays

To determine which BMP favored hASC osteogenesis in vitro, cells were seeded at a density of 4000 cells/cm^2^ (Passage 5) in 6-well plates and grown in DMEM supplemented with 10% FBS (Wisent Bioproducts), antibiotics (100 U/mL penicillin and 25 µg/mL gentamicin), and 1.8 mM calcium chloride (CaCl_2_; Sigma-Aldrich) (referred to as stromal control medium). Cells were grown until approximately 80% confluency (three days of culture). After three days of culture, osteogenic induction was initiated for half of the cultures by supplementing the medium with an osteogenic cocktail composed of 10 nM dexamethasone, 10 nM 1α,25-dihydroxyvitamin D_3_, 50 μM ascorbate-2-phosphate, and 3.5 mM β-glycerophosphate (induction medium). All described supplements were purchased from Sigma-Aldrich. At the same time, for the BMP-treated groups, half of the osteogenically induced hASCs and half of the non-induced controls were treated with 1 nM rhBMP-2 or 1 nM rhBMP-9 (carrier-free CHO, R&D Systems, Minneapolis, MN, USA) for three or six days. The dose was selected based upon previous BMP dose responses performed on human muscle-resident mesenchymal stromal cells [45] and was confirmed with hASCs (Figure S4). The medium was changed three times per week with a freshly prepared BMP supplementation.

### 4.3. Production of the Tissue-Engineered Substitutes

For in vitro and in vivo studies, substitutes were assigned to four experimental groups: stromal substitutes, bone-like substitutes, BMP-9-treated stromal substitutes, and BMP-9-treated bone-like substitutes (Table 2). Tissues were obtained by seeding hASCs at a density of 4000 cells/cm^2^ (Passage 7) in 6-well plates containing a peripheric anchorage device (Whatman paper, Fisher Scientific, Quebec City, QC, Canada), as previously described [23,24,25,61]. Cells were first grown in DMEM supplemented with 10% FBS, antibiotics, and 1.8 mM calcium chloride (CaCl_2_) (basal stromal control medium). Stromal substitutes composed of undifferentiated hASCs were obtained using the basal stromal control medium supplemented with fresh sodium L-ascorbate solution (50 μg/mL; Sigma-Aldrich) at each media change for 21 days of culture. For the bone-like group, hASCs were osteogenically induced by supplementing complete DMEM with an osteogenic cocktail composed of 10 nM dexamethasone, 10 nM 1α,25-dihydroxyvitamin D_3_, and 50 μM ascorbate-2-phosphate (induction medium) after three days of culture including fresh sodium L-ascorbate solution (50 μg/mL) at each media change until the end of the culture. The induction medium was supplemented with 3.5 mM β-glycerophosphate at Day 10 of culture to initiate biomineralization. BMP-treated groups were produced by adding rhBMP-9 to freshly prepared induction or basal stromal control media (1 nM final) after three days and until the end of the culture. Of note, hASC cultures were exposed to a wave-like dynamic movement using an orbital platform (Model 260301F, Ocelot, Fisher Scientific) set at 35 rpm, beginning one day after initial seeding until the end of the culture period. Substitutes were analyzed or implanted after 21 days of culture (18 days of osteogenic induction for the bone-like groups) since this time point allows the best balance between culture time and handling ability [24].

### 4.4. Real-Time Quantitative Polymerase Chain Reactions

Quantitative reverse-transcription polymerase chain reaction (RT-qPCRs) was performed on 2D cell cultures (*n* = 6 samples/condition) and tissue-engineered substitutes (*n* = 4 substitutes/condition, Day 21 of culture). Total ribonucleic acid (RNA) was extracted using the TRIzol reagent (Invitrogen, Carlsbad, ON, Canada) following the manufacturer’s instructions. RNA was then precipitated with isopropanol (Fisher, Mississauga, ON, Canada), centrifuged (12,000× *g*), washed twice with ethyl alcohol (75% *v*/*v*; Fisher), and partially dried in a vacuum centrifuge IA120 SpeedVac concentrator; Thermo Electron Corporation, Madison, WI, USA). The total RNA concentration was quantified by spectrophotometry at 260/280 nm using GeneQuant Pro (Biochrom, Cambridge, UK). Aliquots of RNA (1 µg) were treated with DNase I (1 U/µL), and the first-strand cDNA was synthesized using dNTP (10 mM), Oligo(dT)_12–18_ primer (500 µg/µL), and Superscript^TM^ II reverse transcriptase (200 U/µL) (Invitrogen, Carlsbad, Canada). Quantitative reverse-transcription PCR (RT-qPCR) was carried out using the primers (Qiagen, Montréal, QC, Canada), listed in Table 3, and iQ™ SYBR^®^ Green Supermix (Taq polymerase with SYBR green; BioRad Laboratories, Mississauga, ON, Canada), in triplicate, on an iQ^TM^ Real-Time PCR detection system (BioRad Laboratories, Mississauga, Canada). All assays were normalized to the *Glyceraldehyde-3-phosphate dehydrogenase* (*GADPH*) reference gene, and relative expression levels of the target genes were calculated using the 2^−ΔΔCT^ model [62].

### 4.5. Alkaline Phosphatase Activity Measurement

*ALP* activity was measured after 21 days of culture within media conditioned for 48 h by the substitutes before their in vivo implantation. The activity was evaluated by measuring the hydrolysis rate of p-nitrophenyl phosphate disodium hexahydrate (pNPP) (Santa Cruz Biotechnology, Dallas, TX, USA) as a 5 mM solution in 0.5 M 2-amino-2-methyl-1,3-propanediol/2 mM MgCl_2_ buffer (pH = 10.0). For each condition, fifty microliters of conditioned supernatant were combined with an equal volume of 5 mM pNPP and run in duplicate in a 96-well plate. The *ALP* activity converted substrate pNPP into a p-nitrophenol (pNP) product that was measured at 405 nm using a spectrophotometer after a 30-min incubation period. Results were normalized by total protein content determined by micro-BCA assay quantification (ThermoFisher Scientific, Burlington, ON, Canada) (*n* = 3 substitutes/conditions).

### 4.6. In Vivo Surgical Procedures

**Figure 6:**
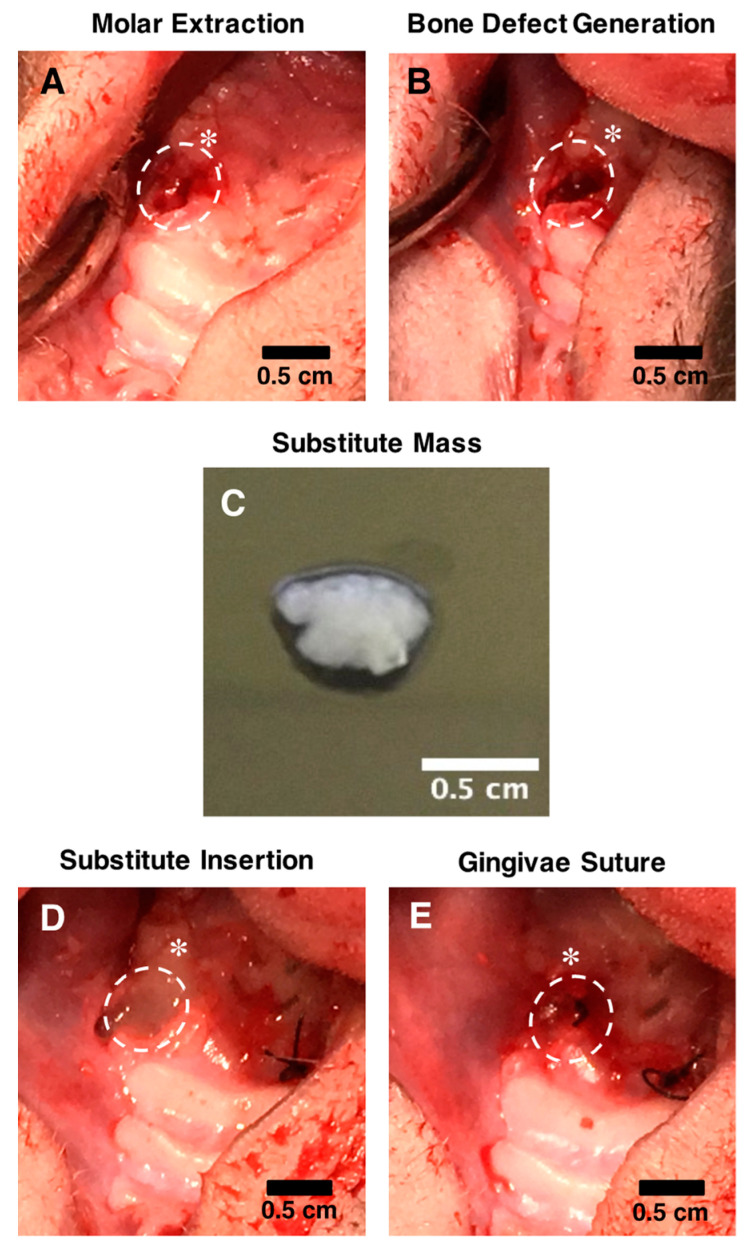
Surgical procedure to assess alveolar bone preservation within a nude RNU rat model. (**A**) Two front molars were extracted bilaterally from the superior maxilla of the rats, and (**B**) 4 mm^3^ holes were subsequently generated using a drill to simulate bone loss following tooth extraction. (**C**) Before grafting, the engineered substitutes were detached from the peripheral anchoring device used during culture and compacted into a tissue mass. (**D**) Bone defects were filled with the substitutes (random allocation) when applicable, and (**E**) gingivae were sutured to maintain the substitutes in the socket. Dotted white circles show the implantation site. The white stars indicate the remaining molar. Scale bars: 0.5 cm.

To evaluate the bone tissue preservation’s potential of the engineered substitutes, alveolar bone defects were generated using a model of immunocompromised NIH-Foxn1^rnu^ rats (21 males, 6–7 weeks old at time of surgery; Charles River, Saint-Constant, QC, Canada) (Figure 6). These animals have a compromised adaptive immune system that tolerates the grafting of human tissues [63]. The surgeries were performed under general anesthesia using ketamine/xylazine intraperitoneal (IP) injection (80/10 mg/kg). Analgesia was provided by IP injection of buprenorphine (0.05 mg/kg) and local injection of lidocaine/bupivacaine (3.5 mg/kg). To generate the defects, two molars on both sides of the superior maxilla were extracted (Figure 6A). A 2 mm diameter drill was then used to simulate alveolar bone loss following tooth extraction and to generate bone defects of approximately 4 mm^3^ (Figure 6B). Before implantation, each type of engineered substitute was washed using sterile PBS and gently detached from the culture plates to generate a compact tissue mass (Figure 6C). The sockets were then filled to the crest with these compacted substitutes (one engineered tissue by socket) or granules of Straumann^®^ BoneCeramic^TM^ biomaterials according to the groups described in Table 2 (for each animal, two different types of substitutes were randomly implanted bilaterally, *n* = 7 for each group) (Figure 6D). Finally, the buccal and palatal gingivae surrounding the implantation site were sutured using a non-traumatic needle and sterile 5-0 nylon suture thread (Mononylon^®^, Ethicon, Bridgewater Township, NJ, USA) (Figure 6E). The sockets of the untreated animals remained empty, and the gingivae were sutured as described for the other groups.

### 4.7. Microcomputed Tomography Imaging and Analysis

A longitudinal study was performed with non-invasive assessments using a microcomputed tomography system with the GE eXplore Locus 80 scanner (GE Healthcare Technologies, Milwaukee, WI, USA). The animal heads were imaged using standard scanning conditions (tube voltage: 80 kV, tube current: 100 µA, exposure time: 90 mS, detectors bin mode: 2 × 2) after four, six, and ten weeks of implantation. Animals were anesthetized by isoflurane inhalation during imaging. The calibration of the standard curve was based on the density of air: −1000 Hounsfield Units (HU), water: 0 HU, and bone (hydroxyapatite): +3000 to +4000 HU. Imaging and bone volume analysis of the 2 mm × 1 mm × 2 mm ellipsoidal regions of interest (ROIs) were performed using the MicroView software (Parallax Innovations, Ilderton, ON, Canada). All analyses were performed using the same threshold level set at 700 (*n* = 7 grafts/condition).

### 4.8. Gingival Healing Evaluation at the Implantation Sites

Macroscopic imaging of the implantation sites was realized post-mortem ten weeks after grafting using a Nikon camera. The quality of gingival healing was evaluated by observing the degree of re-epithelialization at the implantation sites (*n* = 7 implantation sites/condition). Data are expressed as the percentage of implantation sites that featured complete re-epithelialization.

### 4.9. Histological Analysis of Explanted Alveolar Implantation Sites

Histological analysis was performed along the implantation sites ten weeks after grafting. These areas were isolated post-mortem from dissected rat skulls. Briefly, the fresh skulls were fixed in 3.7% formaldehyde overnight and demineralized in 0.6 N hydrochloric acid (HCl) for approximately three weeks at room temperature. Then, these areas were isolated from the maxilla of the skull using a scalpel and processed for paraffin embedding and sectioning. Five micrometer thick cross-sections of the samples were stained following a hematoxylin and eosin protocol. Mosaics were obtained by stitching multiple pictures of the samples captured under brightfield using a Zeiss Observer Z1 inverted microscope (Zeiss) equipped with an AxioCam ICc1 camera.

### 4.10. Statistical Analyses

The mean differences between groups were evaluated by performing one-way or two-way (for data where the time factor is involved) ANOVA with Bonferroni’s multiple comparison post-hoc tests using the GraphPad Prism software (version 8). All data are expressed as the mean ± standard deviation (SD), and differences with a *p* < 0.05 are considered significant.

## 5. Conclusions

In this study, we have shown that hASCs display a better osteogenic response to rhBMP-9 than rhBMP-2 in monolayer cell culture. This was confirmed through the engineering of a 3D bone-like substitute composed of hASCs and cell-assembled extracellular matrix components since rhBMP-9 treatment significantly increased *OSX* pro-osteogenic gene expression and alkaline phosphatase activity after 21 days of culture in vitro. Surprisingly, the long-term rhBMP-9 treatment also significantly increased the gene expression of the pro-angiogenic *angiopoietin-1*. Then, upon grafting, BMP-9-treated bone-like substitutes procured favorable alveolar bone and gingival healing following tooth extraction after ten weeks using a nude rat model. Globally, these substitutes offer promising potential as new biological bone fillers to improve oral bone healing for patients, with the goal of reducing postoperative complications and optimizing dental implant positioning.

## Figures and Tables

**Table 1:** Qualitative evaluation of gingival healing at the implantation sites ten weeks after grafting.

Conditions	Number of Implantation Site Featuring Complete Re-Epithelialization/Total Number of Implantation Sites (% of Re-Epithelialization)
Stromal substitutes	5/7 (71.4%)
Bone-like substitutes	6/7 (85.7%)
BMP-9-treated stromal substitutes	2/7 (28.6%)
BMP-9-treated bone-like substitutes	6/7 (85.7%)
Untreated defects	3/7 (42.9%)
Straumann^®^ BoneCeramic^TM^ biomaterials	5/7 (71.4%)

**Table 2:** List of the experimental groups for in vitro assays and in vivo implantation studies.

Groups	In Vitro Osteogenic Induction	In Vitro BMP Treatment
Stromal substitutes	-	-
Bone-like substitutes	+	-
BMP-9-treated stromal substitutes	-	+
BMP-9-treated bone-like substitutes	+	+
Untreated defects	N/A	N/A
Straumann^®^ BoneCeramic^TM^ biomaterial	N/A	N/A

N/A: Not applicable.

**Table 3:** List of the primer sequences of target genes used for RT-qPCR.

Human Gene	Description	QuantiTect Primer Assay
*ALP*	*Alkaline phosphatase*	Hs_ALPL_1_SG
*RUNX2*	*Runt-related transcription factor 2*	Hs_Runx2_1_SG
*OSX/SP7*	*Osterix transcription factor Sp7*	Hs_Sp7_1_SG
*ANG-1*	*Angiopoietin-1*	Hs_ANGPT1_1_SG
*GAPDH*	*Glyceraldehyde-3-phosphate dehydrogenase*	Hs_GAPDH_1_SG

## Data Availability

The data presented in this study are available on request from the corresponding author.

## Supplementary Materials

The following supporting information can be downloaded at: https://www.mdpi.com/article/10.3390/ijms23063302/s1.
