# Supplementary material for: Preclinical Evaluation of BMP-9-Treated Human Bone-like Substitutes for Alveolar Ridge Preservation following Tooth Extraction
Source: Int J Mol Sci. 2022 Mar 18;23(6):3302. doi: 10.3390/ijms23063302 (PMC8952786; doi:10.3390/ijms23063302)
Supplement: Supplementary file 1 [file ijms-23-03302-s001.zip › ijms-1610508-supplementary.pdf]

Supplementary Materials

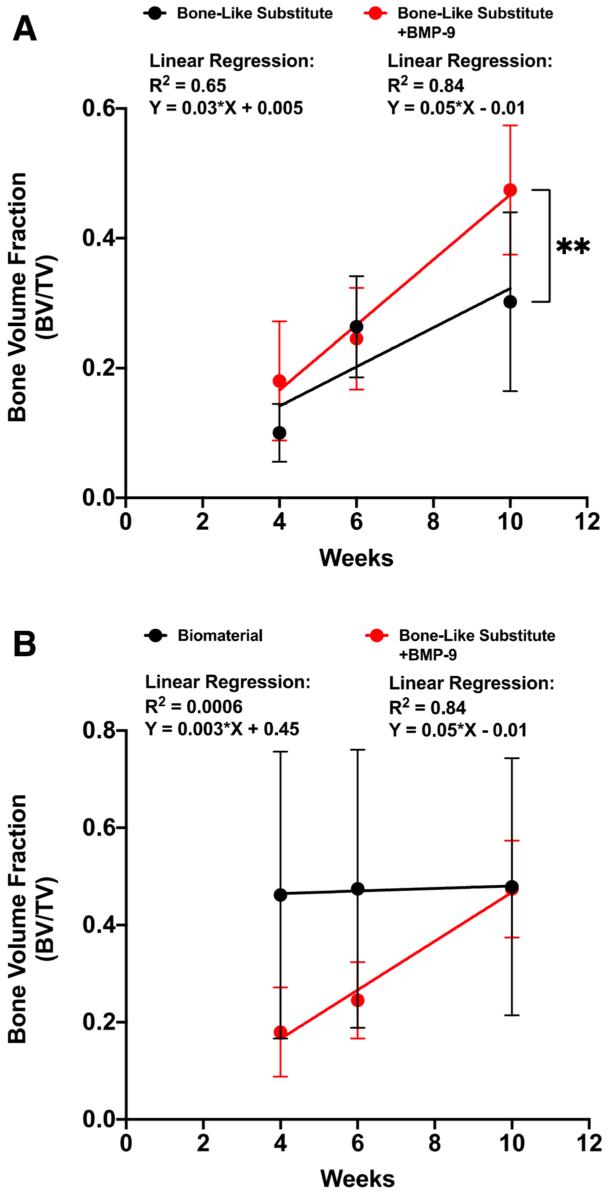

Figure S1. Linear regression curves representing the bone volume fraction evolution over time in vivo. (**A**) A significant difference was observed in bone volume fraction at the implantation site between untreated (black line) and BMP-9-treated (red line) bone-like substitutes after ten weeks. (Two-way ANOVA with Bonferroni's multiple comparison post-hoc test, ** $p < 0.01$). (**B**) Bone-like substitutes treated in vitro with BMP-9 (red line) and the clinical-grade biomaterial (black line) reached similar levels of bone volume fraction after ten weeks of implantation.

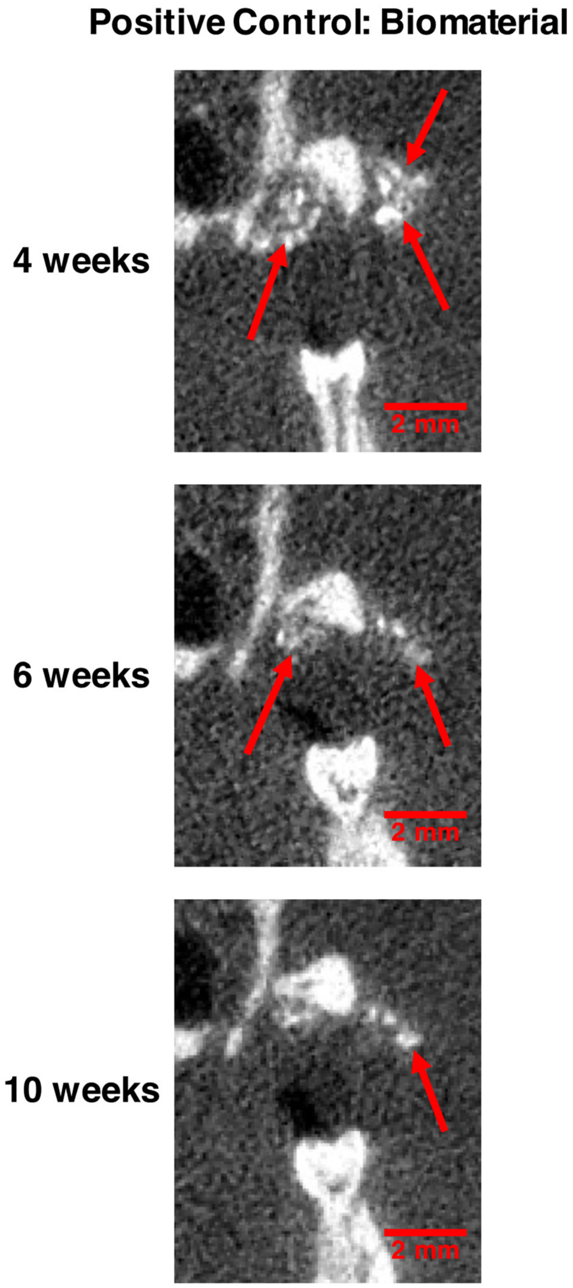

Figure S2. Micro-CT follow-up of the BoneCeramic™ biomaterial resorption after four, six, and ten weeks of implantation using the nude rat model. Coronal plan images show the synthetic biomaterial (red arrows) placed into the empty sockets and maintained using gingival suturing. Three of seven implantation sites showed resorption of the biomaterial over time, as represented here.

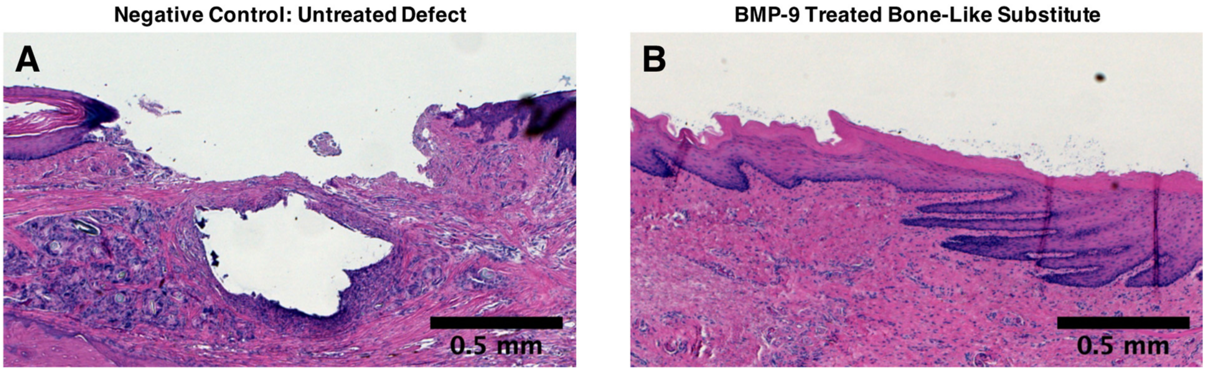

Figure S3. Histological observations of the gingival healing after ten weeks of implantation. (**A**) Representative image of an untreated defect implantation site showing the presence of dehiscence with incomplete gingival coverage. (**B**) Representative image of an implantation site grafted with a BMP-9 treated bone-like substitute displaying complete healing with the presence of stratified gingival epithelium. Scale bars: 0.5 mm.

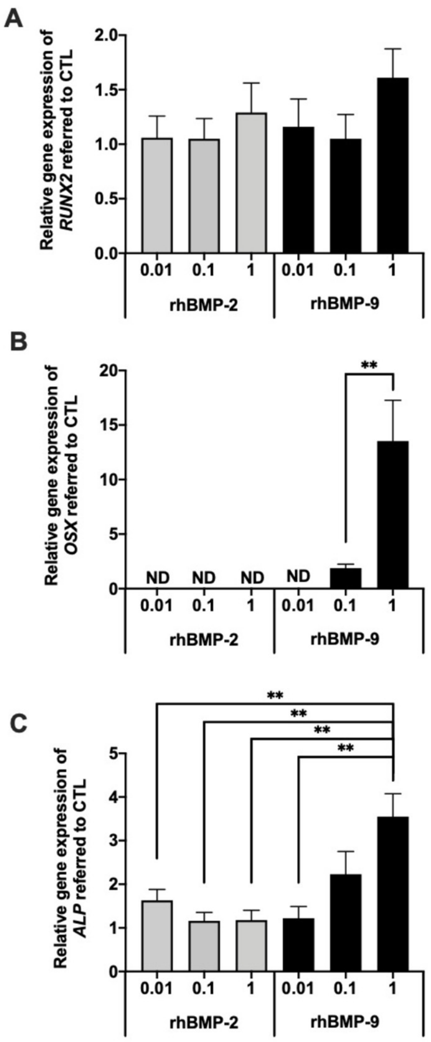

Figure S4. Dose response of rhBMP-2 and 9 on osteogenic gene expression profiles for hASCs in 2D cell culture. (**A**-**C**) Relative gene expression of (**A**) *runt-related transcription factor 2* (*RUNX2)*, (**B**) *osterix* (*OSX*), (**C**) *alkaline phosphatase* (*ALP*) measured by RT-qPCR on non-induced hASCs treated with 0.01, 0.1, or 1 nM of rhBMP-2 or rhBMP-9 for three days. Data were referred to control (CTL) condition which is defined by untreated hASCs cultured in basal stromal control medium for three days. (One-way ANOVA with a Bonferroni's multiple comparison post-hoc test; significant differences between 1 nM rhBMP-9 condition and all other condition: ** $p < 0.01$).
